# Interface-Engineered Copper–Barium Strontium Titanate Composites with Tunable Optical and Dielectric Properties

**DOI:** 10.3390/nano16020096

**Published:** 2026-01-12

**Authors:** Mohammed Tihtih, M. A. Basyooni-M. Kabatas, Redouane En-nadir, István Kocserha

**Affiliations:** 1 Institute of Energy, Ceramics, and Polymer Technology, University of Miskolc, Egyetemváros, H-3515 Miskolc, Hungary; 2 Department of Precision and Microsystems Engineering, Delft University of Technology, Mekelweg 2, 2628 CD Delft, The Netherlands; 3 Department of Nanotechnology and Advanced Materials, Graduate School of Applied and Natural Science, Selçuk University, Konya 42030, Turkey; 4 3IT, Faculty of Engineering, 2500 Bd de l’Université, Sherbrooke, QC J1N 3C6, Canada

**Keywords:** Barium strontium titanate ceramics, BST/Cu composite system, spark plasma sintering, optical bandgap reduction, electrical conductivity, dielectric permittivity

## Abstract

We report the synthesis and multifunctional characterization of copper-reinforced Ba_0.85_Sr_0.15_TiO_3_ (BST) ceramic composites with Cu contents ranging from 0 to 40 wt%, prepared by a sol–gel route and densified using spark plasma sintering (SPS). X-ray diffraction and FT-IR analyses confirm the coexistence of cubic and tetragonal BST phases, while Cu remains as a chemically separate metallic phase without detectable interfacial reaction products. Microstructural observations reveal abnormal grain growth induced by localized liquid-phase-assisted sintering and progressive Cu agglomeration at higher loadings. Scanning electron microscopy reveals abnormal grain growth, with the average BST grain size increasing from approximately 3.1 µm in pure BST to about 5.2 µm in BST–Cu40% composites. Optical measurements show a continuous reduction in the effective optical bandgap (apparent absorption edge) from 3.10 eV for pure BST to 2.01 eV for BST–Cu40%, attributed to interfacial electronic states, defect-related absorption, and enhanced scattering rather than Cu lattice substitution. Electrical characterization reveals a percolation threshold at approximately 30 wt% Cu, where AC conductivity and dielectric permittivity reach their maximum values. Impedance spectroscopy and equivalent-circuit analysis demonstrate strong Maxwell–Wagner interfacial polarization, yielding a maximum permittivity of ~1.2 × 10^5^ at 1 kHz for BST–Cu30%. At higher Cu contents, conductivity and permittivity decrease due to disrupted Cu connectivity and increased porosity. These findings establish BST–Cu composites as tunable ceramic–metal systems with enhanced dielectric and optical responses, demonstrating potential for specialized high-capacitance decoupling applications where giant permittivity is prioritized over low dielectric loss.

## 1. Introduction

Ba_1−x_Sr_x_TiO_3_ (BST), a highly versatile ABO_3_ perovskite oxide, is a material of critical importance in modern electronics due to its exceptional ferroelectric and dielectric properties [1,2,3]. Its high relative permittivity and strong electrical tunability make it an ideal candidate for high-frequency microwave devices, high-density energy storage capacitors, and integrated electronic circuits. However, the practical utility of pristine BST ceramics is often constrained by two major factors first, BST is a wide-bandgap semiconductor (typical bandgap > 3.2 eV), consequently; its photoabsorption is limited exclusively to the ultraviolet (UV) region. This characteristic severely restricts its potential in emerging visible-light-driven applications [4,5], such as photocatalysis and photosensors. Second, while BST exhibits high permittivity, the drive toward miniaturization necessitates even greater dielectric constants that are stable across a wide range of temperatures and frequencies [6]. Recent studies have explored the incorporation of metallic phases into ceramic matrices to address this limitation and tailor their optical and electrical properties [7,8,9,10]. Copper (Cu), in particular, has been identified as a suitable addition to the composite due to its ability to reduce the bandgap and enhance electrical conductivity in perovskite ceramics [11,12]. Moreover, spark plasma sintering has emerged as a promising technique for fabricating dense ceramic composites with controlled microstructures and enhanced properties [13].

To circumvent these material-specific limitations, engineering strategies based on the formation of ceramic-metal composites are highly attractive. While chemical doping (substituting host ions with a foreign element) is one method for property modification, it often requires high temperatures and prolonged processing to achieve a solid solution, and the resulting electronic changes can be subtle. The present study, conversely, focuses on the creation of a two-phase composite consisting of BST grains reinforced with discrete metallic Cu particles. This method, facilitated by a Sol–Gel-derived powder followed by rapid Spark Plasma Sintering, is specifically chosen because it favors the formation of sharp BST/Cu interfaces while minimizing bulk chemical reaction, thus preserving the distinct phases.

The strategic choice of metallic Cu is motivated by the pronounced functional benefits that arise from these ceramic-metal interfaces:

1. Enhanced Dielectric Response: The introduction of a highly conductive metallic phase (Cu) into the insulating BST matrix creates numerous internal boundaries. This heterogeneous structure leads to a buildup of charge carriers at the interfaces when subjected to an external electric field, a phenomenon known as Maxwell–Wagner (MW) interfacial polarization. This MW effect is known to dramatically increase the effective relative permittivity of the composite far beyond the intrinsic value of the BST matrix itself.
2. Extended Optical Absorption: The presence of metallic Cu provides a distinct mechanism for visible light interaction. Cu nanoparticles are renowned for generating Localized Surface Plasmon Resonance (LSPR), which results in strong, tunable light absorption across the visible spectrum. By utilizing both the LSPR effect and the formation of new electronic interface states at the BST/Cu boundary, the composite system is expected to exhibit a significant reduction in its effective optical band gap and enhanced absorption in the solar spectrum.

In this study, we report the synthesis and characterization of Ba_0.85_Sr_0.15_TiO_3_/Cu (BST-Cu) composites with varying Cu content (0–40 wt%). The structural, optical, and electrical properties of these composites were systematically investigated, with a focus on understanding the role of Cu in modifying the bandgap, conductivity, and dielectric behavior of BST. The results demonstrate that BST-Cu composites exhibit tunable multifunctional properties, making them suitable for a wide range of applications, including multilayer ceramic capacitors (MLCCs), optoelectronic devices, and micro-optical electro-mechanical systems (MOEMS).

## 2. Materials and Methods

### 2.1. Synthesis of Ba_1−x_Sr_x_TiO_3_ Ceramics

The pure and Sr-modified BaTiO_3_ powders were prepared through the sol–gel method [14,15] using Barium acetate trihydrate (Ba (CH_3_CO_2_)_2_·3H_2_O) (99.9% purity), Strontium acetate (C_4_H_6_O_4_Sr) (99% purity) and Titanium alkoxide Ti [OCH(CH_3_)_2_]_4_ (97%, purity) as primary precursors, while introducing lactic acid and acetic acid as pivotal stabilizing agents, a strategic evolution inspired by recent investigations into solution chemistry optimization. The process commences with the dissolution of titanium isopropoxide in a precisely calibrated mixture of water and acetic acid, maintaining a delicate equilibrium at 70 °C while utilizing an agitation mechanism at 300 rpm, a parameter refined based on fluid dynamics modeling [16,17]. Generally, the raw materials used in the sol–gel process were metal alkoxides and acetates. The specification of raw materials is given in Table 1.

Lactic acid and acetic acid were employed as complementary chelating and stabilizing agents in the sol–gel process. Acetic acid moderates the hydrolysis rate of titanium isopropoxide by forming acetate complexes, preventing premature precipitation, while lactic acid acts as a stronger bidentate chelator, stabilizing Ti^4+^ species and promoting homogeneous gel formation. The selected lactic acid/Ti molar ratio (~1:1) has been reported to suppress secondary phase formation and improve powder homogeneity in BaTiO_3_-based sol–gel systems. Similar formulations were shown to enhance phase purity and control particle morphology in perovskite oxides [18,19]. In preliminary trials, this formulation yielded transparent sols and phase-pure BST after calcination, whereas lower lactic acid contents resulted in partial segregation of TiO_2_.

Several Ba_1−x_Sr_x_TiO_3_ compositions (x = 0, 5, 12.5, 15, 20, and 30%) were synthesized during preliminary optimization to identify a host matrix that exhibits stable phase coexistence, good sinterability, and reproducible dielectric behavior. Based on these trials, Ba_0.85_Sr_0.15_TiO_3_ was selected as the optimal host matrix for the composite system. This specific composition was chosen because it crystallizes in a perovskite structure, allowing for the stable coexistence of cubic and tetragonal phases at room temperature. This provides a high and stable dielectric response, which is ideal for investigating the interfacial effects induced by subsequent metallic Cu addition. While the broader range of x-values establishes the processing window, the present study focuses exclusively on the Ba_0.85_Sr_0.15_TiO_3_/Cu system to isolate the influence of the metallic phase on the optical and electrical properties of a consistent ceramic matrix [2]. Following the selection of the host composition, a colloidal solution of Titanium alkoxide was prepared (Solution B). Preparing 500 mL of 1 mol/L titanium sol requires 143.5 g of Titanium alkoxide, 22.5 g of lactic acid, and 300 mL of distilled water. A 1% excess of Titanium was added to the lactic acid solution to account for losses of Ti due to hydrolysis and the sol filtration operation. Introducing titanium alkoxide into the lactic acid solution, stirred at 70 °C, yields a white, milky precipitate immediately. The obtained mixture, subjected to continuous stirring for about 24 h, gradually passes from a white solution to a completely transparent sol (Solution B). The third step involves mixing the obtained solutions (A and B). These solutions are mixed in stoichiometric proportions, according to the chemical formula Ba_1−x_Sr_x_TiO_3_ (x = 0, 0.05, 0.125, 0.15, 0.2, and 0.3) with stirring for 5 min, to ensure the homogeneity of the final solution. The destabilization of this solution is ensured by evaporation of the solvent in a programmable oven at 80 °C for 48 h (Step 4). The obtained xerogel is ground in an agate mortar to break up the powder agglomerates and increase its responsiveness. The powders, after grinding, were calcined in air in a programmable furnace at 950 °C for 3 h.

### 2.2. Preparation of BST-Cu and the Spark Plasma Sintering Process

**Figure 1:**
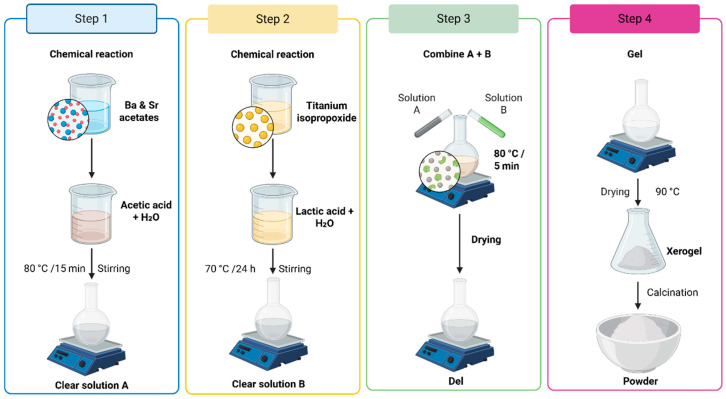
Ba_1−x_Sr_x_TiO_3_ (x = 0.0–0.3) synthesis flowchart summarizing the preliminary screening of host compositions.

The obtained Barium strontium titanate (BST) powders were mixed with high-purity copper powders (Cu, purity: 99.8%, average particle size: 5 µm, Sigma Aldrich, Germany) (as shown in the schematic Figure 1). First, the BST powders were mixed with Cu content ranging from 0 to 40 wt% by ball milling using ethanol in a resin container for 12 h, with zirconia balls serving as the grinding media. Subsequently, the slurry was dried, and the resulting mixed powders were extracted. To densify the BST–Cu composite powders, spark plasma sintering was performed at the Tallinn University of Technology, Department of Mechanical and Industrial Engineering, Tallinn, Estonia. The SPS process utilized a graphite die assembly with an inner diameter of 20 mm, lined with graphite foil to facilitate sample removal and ensure uniform current distribution. The temperature was monitored via an optical pyrometer focused on a bore in the graphite die surface, approximately 2 mm from the sample. A constant heating rate of 100 °C/min was maintained to ensure rapid through-heating while suppressing excessive grain growth. After reaching T_F_, a uniaxial pressure of 50 MPa was applied, and a dwelling time of 15 min was maintained for sintering, after which the pressure was released, and the samples were cooled naturally within the vacuum chamber. In a typical processing cycle, powder (2–2.6 g) was loaded into the die. The BST/Cu composite system powders were initially heated under vacuum to 600 °C within 3 min using a pulsed direct current (DC) power source characteristic of Spark Plasma Sintering. This rapid initial stage was employed to minimize pre-sintering oxidation of the Cu phase. Above this temperature, a heating rate of 100 °C/min was applied until the final sintering temperature (TF = 950 °C) was reached. The sintering temperature was maintained at 950 °C for both pure BST and BST/Cu composite systems to ensure a valid comparison between the host matrix and the composites. While pure BST typically requires higher conventional sintering temperatures (>1200 °C), the application of SPS at 950 °C was chosen specifically to prevent the melting and excessive coalescence of the metallic Cu phase (m.p. ~1085 °C). The simultaneous application of a uniaxial pressure of 50 Mpa during the SPS process compensates for the lower temperature by providing an additional driving force for densification through grain boundary sliding and plastic deformation, resulting in high density in both pure ceramic and metal-reinforced samples. The temperature was monitored and regulated using an optical pyrometer focused on the surface of the graphite die. After reaching TF, a uniaxial pressure of 50 Mpa was applied, and a dwelling time of 15 min was maintained for sintering. The SPS parameters (950 °C, 50 Mpa) were selected based on a combination of preliminary densification trials and literature reports on BaTiO_3_–metal composites. Lower temperatures (<900 °C) resulted in insufficient densification, while higher temperatures promoted excessive Cu coalescence. The applied pressure of 50 Mpa ensured rapid densification while suppressing excessive grain growth, consistent with prior studies on SPS of BST-based ceramics.

## 3. Results and Discussion

### 3.1. Phase Analysis Using XRD

**Figure 2:**
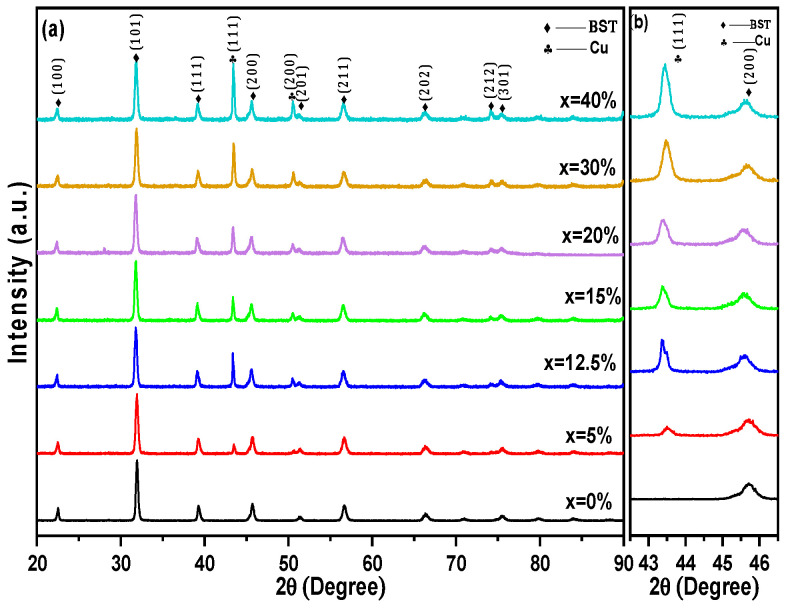
(**a**) XRD pattern of BST-Cux (x = 0–40%) ceramic-metal composites (**b**) Shifting of the peaks BST(200) and Cu(111).

The XRD patterns of BST-Cu_x_ (x = 0, 5, 12.5, 15, 20, 30, and 40%) composites sintered at a low temperature of 950 °C, tested at room temperature, are shown in Figure 2a. All peaks were attributed to either the BST phase or Cu without any impurities being observed, suggesting no reaction occurred between BST and Cu during the SPS process. To address the potential overlap between metallic Cu and copper oxide reflections, the XRD patterns were carefully inspected for the characteristic peaks of Cu_2_O (e.g., at 2θ~36.4°) and CuO (e.g., at 2θ~35.5° and 38.7°). No such reflections were detected above the background noise in any of the composite samples. The assignment of the peak at ~43.3° to metallic Cu(111) is further supported by the use of high-purity metallic precursors and the Spark Plasma Sintering conditions, which were conducted under a vacuum of ~10^−2^ mbar and in a reducing graphite environment. These processing parameters significantly suppress the oxidation of the metallic phase, ensuring that copper remains in its elemental metallic state within the BST/Cu composite system. To quantify the relative proportions of the constituent phases, a semi-quantitative analysis was performed using the Reference Intensity Ratio (RIR) method based on the integrated intensities of the most prominent reflections for the tetragonal BST (P4mm), cubic BST (Pm-3m), and metallic Cu (Fm-3m) phases. The calculated phase fractions for the Ba_0.85_Sr_0.15_TiO_3_ host matrix revealed a coexistence of approximately 65% cubic and 35% tetragonal phases at room temperature. For the composite samples, the extracted weight percentages of metallic Cu were found to be 9.8 ± 0.5%, 19.4 ± 0.7%, and 38.9 ± 1.1% for the 12.5, 20, and 40 wt% nominal loadings, respectively. These values align closely with the initial experimental compositions, confirming that no significant phase loss or unexpected chemical reactions occurred during the Spark Plasma Sintering process. Furthermore, the diffraction peaks of BST in the composites did not shift, indicating that the copper was not incorporated in the perovskite structure [20]. As expected, the relative diffraction intensities of the Cu reflections compared to the ones of BST increased with the increase in Cu content (Figure 2b). The evolution of the XRD peaks confirms successful control of composition during the sintering process. Additionally, the crystal structure of BST-Cu samples has been proven to be a multiphase coexistence of cubic and tetragonal phases at room temperature. The base Ba_0.85_Sr_0.15_TiO_3_ ceramic crystallizes in a perovskite structure with coexisting tetragonal (space group P4mm) and cubic (space group Pm–3m) phases at room temperature. The refined lattice parameters are consistent with values reported in the literature for Sr-modified BaTiO_3_.

### 3.2. FT-IR Analysis

**Figure 3:**
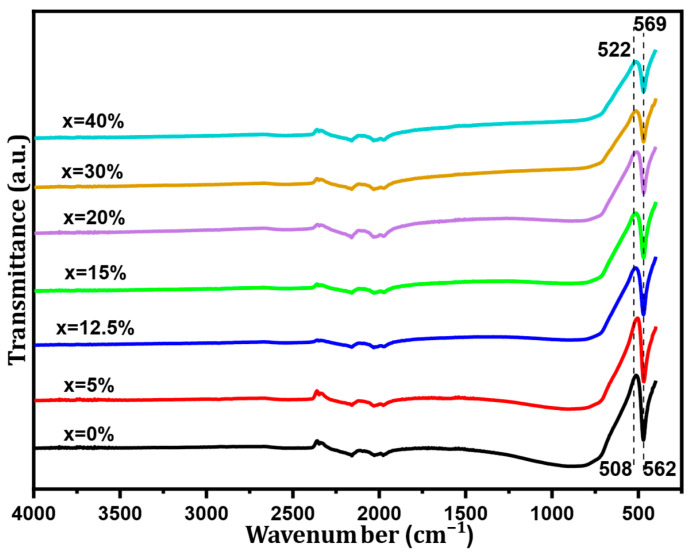
FT-IR spectra of the BST-Cu_x_ (x = 0, 5, 12.5, 15, 20, 30, and 40%) composite sample.

The Fourier transform infrared spectroscopy (FT-IR) spectra of BST-Cux (x = 0, 5, 12.5, 15, 20, 30, and 40%) composites in the wavenumber range from 4000 to 450 cm^−1^ are displayed in Figure 3. These spectra show three sets of absorption bands. The first one is characterized by a wide band in the low-frequency range from 460 cm^−1^ to 723 cm^−1^, associated with the vibrations of the TiO_6_ octahedron. Moreover, all the samples show the molecular fingerprint of BST as revealed by the Ti-O and Ti-O-Ti bonds between 460 cm^−1^ and 601 cm^−1^. The absorption peaks for the same mode BST-Cux (x = 0, 5, 12.5, 15, 20, 30, and 40%) were observed at around 470–480 cm^−1^. Incorporating Sr into the BaTiO_3_ lattice moved the characteristic peak of Ti-O to higher energy values. In our samples, the substitution of Ba^2+^ ions by Sr^2+^ ions influenced the binding distance between ions and atoms [21,22]. On the other hand, an apparent band absorption was observed at approximately 1061 cm^−1^, indicating that it can be attributed to interfacial electronic states and vibrational modes at the BST/Cu boundaries. In addition, the bands observed in the region from 1430 cm^−1^ to 1550 cm^−1^ could be attributed to symmetrical and antisymmetric vibrations (stretching of carboxyl groups bound to barium and/or titanium (COO^−^)). Therefore, our results are in agreement with those reported in the literature [21]. The observations on infrared spectra are in agreement with those revealed by XRD analysis.

### 3.3. SEM Investigation

**Figure 4:**
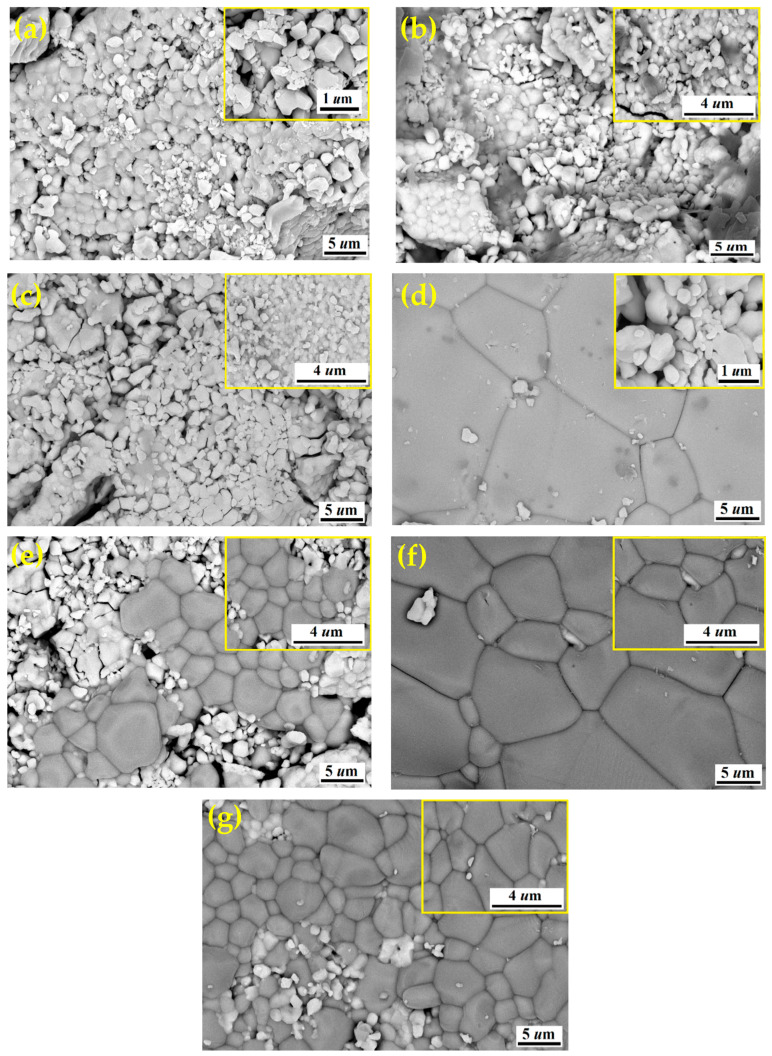
FESEM micrographs of the ceramic samples (**a**) BST, (**b**) BST-Cu5%, (**c**) BST-Cu12.5%, (**d**) BST-Cu15%, (**e**) BST-Cu20%, (**f**) BST-Cu30%, (**g**) BST-Cu40%.

The SEM micrographs of the BST-Cux composites with different Cu content (x = 0, 5, 12.5, 15, 20, 30, and 40 wt%) are shown in Figure 4. The shapes of the grains were also irregular. The interfacial area between BST and Cu increased with increasing Cu content, thereby increasing the amount of the interfacial region. It was seen in Figure 4d–g that abnormal grain growth was clearly observed. The normal grain size of pure BST was about 3.1 µm, while the abnormal grain size of BST-Cu composites was about 3.1–5.2 µm. Abnormal grain growth was generally known to occur when particle shape was angular in the presence of a liquid phase during sintering [23]. It is indicated that the low eutectic liquid phase formed between Cu and BST during sintering promoted grain growth [24]. In Figure 4c, many big spheroidal copper particles stood in the BST matrix. That is, the Cu is agglomerated with an increasing amount of Cu. The coalescence of Cu may be caused by pores, which also contribute to the decrease in relative density of BST-Cu composites with the rising amounts of Cu. The spheroidal copper particles on the surface of the BST matrix (Figure 4d–g) were identified as Cu by EDS analysis. Following the methodology used for ceramic-metal composite systems [25], high-resolution SEM was used to separately demonstrate the perovskite grain structure (characterized by angular grains and defined boundaries) and the non-perovskite regions (characterized by the brighter, more ductile metallic copper phase). The sharp transition between these regions confirms that no intermediate reaction layer formed, which is consistent with the interfacial engineering approach used in this study. Figure 4a shows an agglomeration with high porosity in the synthesized sample. Agglomeration is caused by incomplete dispersion during sol–gel drying and calcination, resulting in strong interparticle interactions and cluster formation, not by a high surface-to-volume ratio. The abnormal grain growth observed in BST–Cu composites is attributed to the transient formation of a Cu-rich low-melting interfacial liquid phase during SPS. Although no in situ thermal or microscopic analysis was performed, the rapid heating rate, applied pressure, and the relatively low melting temperature of Cu strongly favor localized liquid-phase-assisted mass transport. Importantly, XRD and EDS analyses revealed no evidence of reaction products or interfacial reaction layers (e.g., CuO, Cu_2_O, or Ba–Cu oxides), indicating that Cu remains chemically separate from the BST matrix.

Similarly, Figure 4b shows the SEM micrographs of the BST–Cu5% material, revealing a rounded grain morphology. Table 2 presents the variation in BST grain size as a function of Cu content. Grain size was determined using ImageJ (1.53j) by measuring the equivalent circular diameter of more than 200 BST grains per sample, explicitly excluding all Cu particles, and the reported values represent the BST mean grain size with a typical standard deviation of ±0.3 µm. An increase in Cu content promotes local liquid-phase formation during SPS, facilitating abnormal grain growth in certain BST regions; however, at Cu contents above 30 wt%, the microstructure becomes more heterogeneous, and the overall apparent BST grain size does not increase monotonically due to enhanced Cu agglomeration and porosity. This behavior is consistent with the XRD observations, which indicate increased phase separation at higher Cu loadings. Although BST–Cu30% appears coarser in SEM compared with BST–Cu40%, this difference is attributed to 2D sectioning effects and Cu agglomeration rather than a genuine reduction in BST grain size. To quantitatively evaluate the densification behavior, the relative density of the composites was measured using Archimedes’ method. The relative density decreased from 98.2% for pure BST to 92.4% for the BST-Cu40% composite. This trend confirms that while the localized formation of a low-eutectic Cu-based liquid phase at the SPS temperature (950 °C) promotes the observed abnormal grain growth, the simultaneous agglomeration of Cu particles and the mismatch in thermal expansion coefficients lead to increased interfacial porosity. This quantitative increase in porosity (from <2% to ~7.6%) correlates with the degradation of dielectric performance observed at higher loadings.

Quantitative EDS point analysis was performed to distinguish the perovskite ceramic phase from the metallic phase and verify stoichiometric consistency. In the perovskite regions, the atomic percentages of Ba, Sr, Ti, and O were found to align with the theoretical ABO_3_ structure. Specifically, the A-site sum (Ba + Sr) and the B-site (Ti) maintain a near-perfect 1:1 ratio, while the oxygen content satisfies the 3-fold requirement relative to titanium (O/Ti ≈ 3.0). Conversely, in the metallic inclusions, the Cu content is explicitly reported as the dominant phase, with oxygen levels remaining negligible. These quantitative findings, summarized in Table 3, demonstrate the absence of significant oxygen incorporation into the copper phase. Following the stoichiometric verification methodology of Sheeraz et al. [26], this confirms the successful formation of a BST/Cu composite system with distinct, non-reactive interfaces where copper remains in its metallic Cu^0^ state, satisfying the 1:1:3 stoichiometric balance required for the perovskite host.

**Figure 5:**
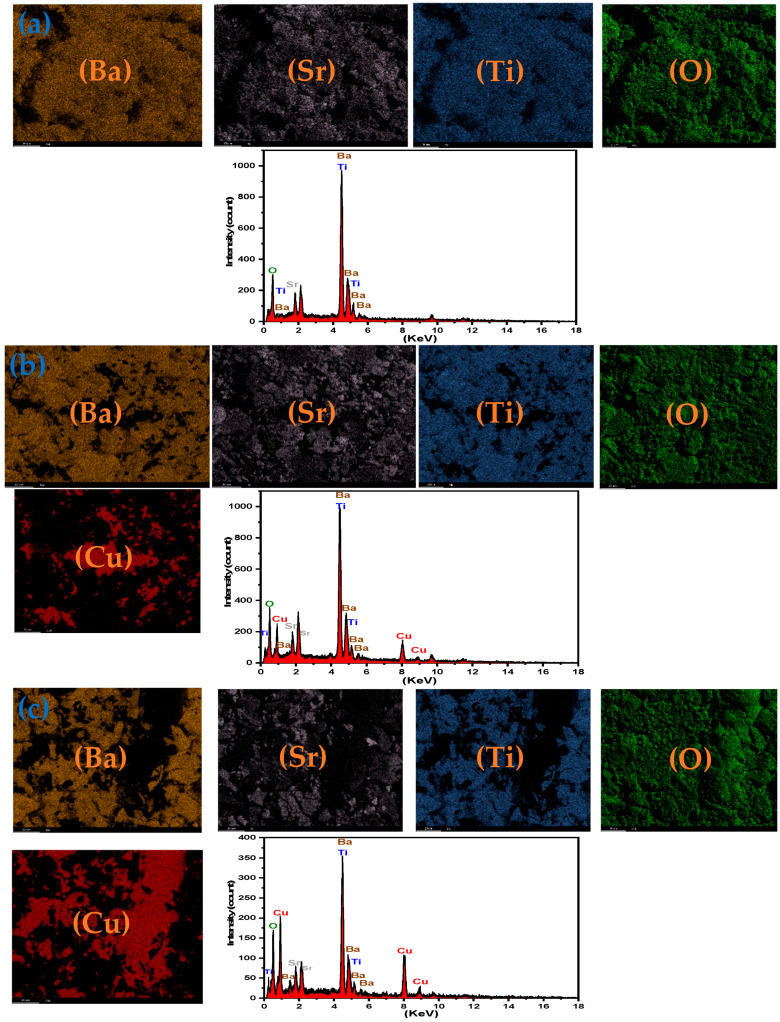
EDS and elemental mapping of (**a**) BST, (**b**) BST-Cu15%, (**c**) BST-Cu40%.

The EDS spectra and elemental mapping images for BST–Cu composite ceramics with the marked spectral positions of the individual elements are displayed in Figure 5. Qualitative EDS analysis confirms the expected stoichiometry of Ba, Sr, Ti, O, and Cu, without any detectable foreign contaminants. The elemental maps further verify the spatial distribution of BST and Cu phases within the microstructure.

The presence of oxygen within Cu-rich regions originates from two contributing factors: (i) native surface oxidation of Cu, forming thin Cu_2_O/CuO layers during polishing and subsequent air exposure, and (ii) the comparatively large interaction volume of EDS (approximately 1–2 μm), which permits the detector to sample underlying BST grains even beneath Cu particles. The amount of Cu oxide that forms under these conditions remains below the XRD detection threshold (typically ~2–3%), which explains the absence of CuO or Cu_2_O reflections in the diffraction patterns.

### 3.4. Optical Characteristics

**Figure 6:**
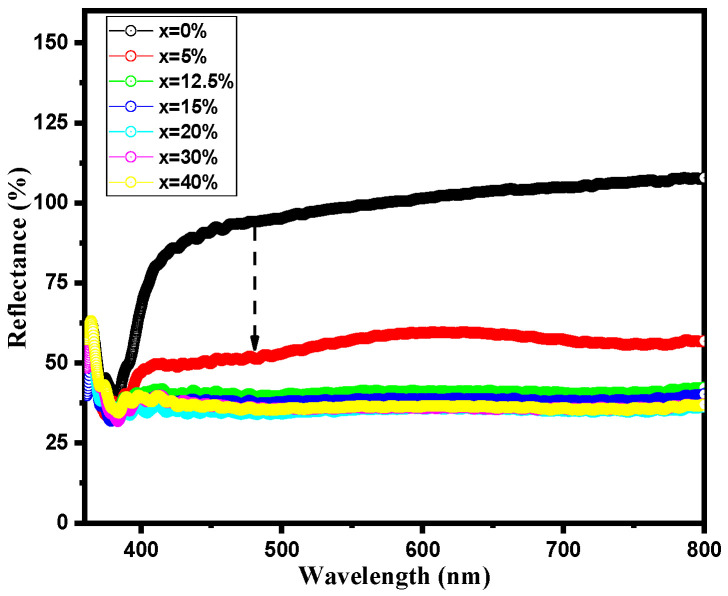
Diffused reflectance spectra of BST-Cux (x = 0–40%) samples.

The correlation between crystalline structure and physical properties in perovskite materials is very delicate. Small structural changes can induce significant variations in their physical and optical properties. The optical behavior is analyzed employing UV-Visible absorption spectroscopy. Diffused reflectance UV–vis spectra of pure and BST-Cu composites measured in the range of 350–800 nm are shown in Figure 6. Samples with a high copper content exhibit an intriguing behavior of absorbing visible photons in the region above 400 nm, with a maximum absorption of 45%.

Although the samples exhibit enhanced absorption in the visible range with increasing Cu content, the XRD patterns show no shift in the characteristic BST peaks, confirming that Cu does not substitute into the BST lattice and instead remains as a distinct metallic phase. Therefore, the observed changes in optical response arise from interfacial effects, defect-related absorption, and scattering from the BST–Cu phase boundaries rather than any substitution-driven modification of the BST electronic band structure. The optical band gap energy (E_g_) of BST-Cu_x_ (x = 0–40%) samples was determined using the Kubelka-Munk (K–M) method, which was employed to extract Eg values with high accuracy [27].

**Figure 7:**
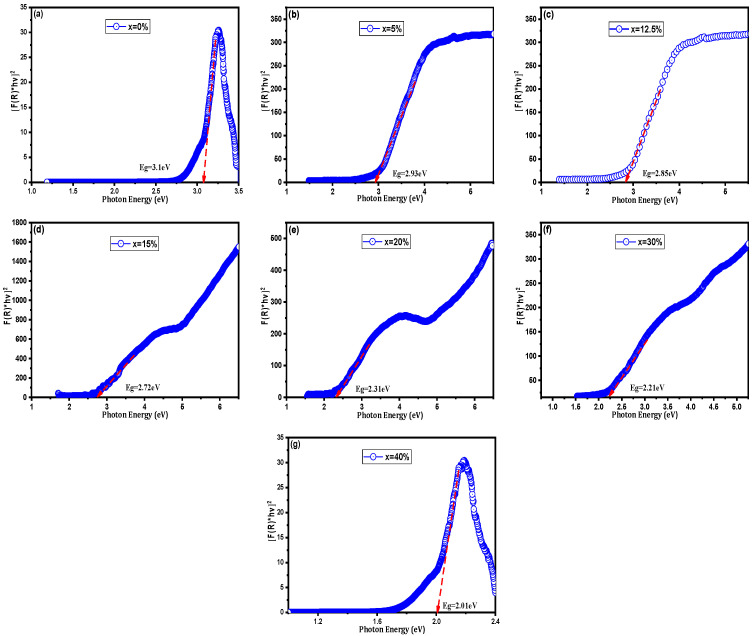
Band gap energy from Tauc plot of (**a**) BST, (**b**) BST-Cu5%, (**c**) BST-Cu12.5%, (**d**) BST-Cu15%, (**e**) BST-Cu20%, (**f**) BST-Cu30%, and (**g**) BST-Cu40%.

It is important to note that the bandgap values extracted via the Tauc method for these composites represent an effective optical bandgap rather than the intrinsic electronic bandgap of the BST lattice. In ceramic-metal systems, the introduction of metallic Cu creates a high density of interfacial states and localized surface plasmon resonance (LSPR) effects. These phenomena enhance absorption in the visible spectrum, causing a significant red shift in the apparent absorption edge. Especially as the system approaches the percolation threshold, the reported values reflect the collective optical response of the composite rather than a structural narrowing of the ceramic’s fundamental gap. As demonstrated in Figure 7 and listed in Table 4, the E_g_ values of BST-Cux (x = 0, 5, 12.5, 15, 20, 30, and 40%) obtained by extrapolating the linear part to the horizontal axis are in the range of 3.10 and 2.01 eV, which are considerably lower than that of the BST (3.10 eV). Among them, the apparent optical absorption edge shift in BST-Cu30% (Figure 7f) is the most evident and is even more significant than those of other photovoltaic perovskite ceramics [28,29]. These optical behaviors can be explained by the newly emerging electronic states of the valence band maximum (VBM) and the next highest energy band above the unoccupied valence band (conduction band minimum, CBM), generated by Sr^2+^ cation substitution. In BST, the VBM is around the O 2p orbital, which slightly interacts with the Ti 3d and Ba 6p orbitals, whereas the CBM is around the Ti 3d orbital [30]. The position of the conduction band is influenced by interfacial effects between BST and Cu; these interactions at the phase boundaries can lead to a shift in the effective conduction band edge [31]. The VBM is localized around the Ti 3d orbital when the copper phase is introduced. Due to the interfacial effects between BST and Cu, and since Cu does not substitute into the BST lattice, Cu-related electronic states cannot be responsible for the observed activation energies. Hence, there is a downward shift in the conduction band edge into the band gap, reducing E_g_. Moreover, according to Choi’s report [32], in addition to interfacial electronic states, the optical band gap may be related to the lattice distortion caused by the ion substitution. The lattice distortion increases as the radius of the added metallic particles decreases, leading to a rearrangement of the molecular orbitals and a reduction in the band gap. Since the samples consist of insulating BST and metallic Cu, the Kubelka–Munk model yields only an apparent absorption edge rather than a true semiconductor bandgap. Although direct spectroscopic evidence (XPS or EPR) was not obtained in this study, the presence of oxygen-vacancy-related defect states is strongly supported by the low activation energies (0.13–0.18 eV) extracted from Arrhenius analysis, which are consistent with oxygen-vacancy-assisted hopping in reduced BaTiO_3_ systems. Furthermore, SPS processing under reduced oxygen partial pressure is well known to induce oxygen vacancies in perovskite oxides, contributing to sub-bandgap optical absorption and enhanced conductivity [33,34]. The decrease in the apparent bandgap may result from increased scattering, interface-induced absorption, or the presence of defect states. Thus, the extracted Eg values should be interpreted qualitatively rather than as intrinsic band gaps.

According to the experimental data analysis, copper may be the preferred element to reduce the band gap of the BST material. These results indicate that we can effectively reduce the band gap of BST ceramics by copper reinforcement, thereby improving their visible and ultraviolet absorption properties. We believe optimizing the Cu concentration can further modify the band gap of the BST ceramic material. Consequently, all the above-presented results indicate that the optical properties of BST-Cu_x_ (x = 5, 12.5, 15, 20, 30, and 40%) ceramics can be regulated, demonstrating that the synthesized ceramics are promising candidates for high-performance multilayer ceramic capacitors.

### 3.5. Electrical Conduction Mechanism in BaTiO_3_–Cu Composites

**Figure 8:**
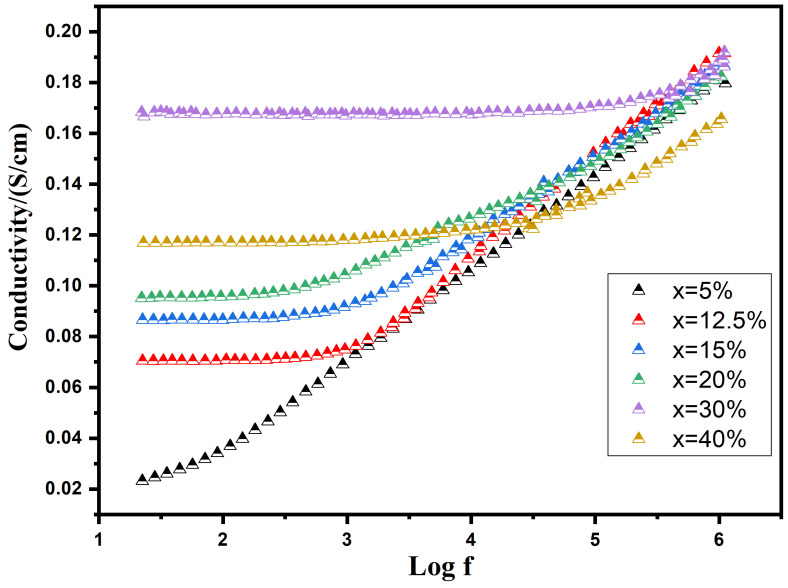
Frequency-Dependent modulation of AC Conductivity (σ′) with varying Copper additions at Room Temperature.

The graph in Figure 8 illustrates the change in the real part of AC conductivity (σ′) with frequency at room temperature, considering various additions of Cu. AC conductivity gradually increases with rising Cu concentrations up to 30 wt%, followed by a decline as the Cu content continues to rise. This shift in conductivity is attributed to fluctuations in the concentration of charge carriers within the composites. As the Cu content surpasses a specific threshold (percolation threshold), the concentration of charge carriers decreases due to the emergence of the Cu liquid phase during sintering. This phenomenon aligns with the apparent density variations observed in composites with varying Cu contents. The percolation threshold for BST-Cu_x_ is identified at a concentration of 30 wt%. At lower frequencies, a distinct plateau, more pronounced in samples with higher Cu contents than lower ones, reflects the direct current conductivity ($\sigma_{dc}$). Conversely, a dispersive region, known as the “universal dielectric response” (UDR), becomes evident at higher frequencies. This can be approximately characterized as follows [35]:

(1) $$f_{m}=\frac{1.8\times 10^{12}}{\varepsilon \rho }$$

In this context, the dispersive frequency fm is defined as the point at which the transition from the plateau-type region of direct current conductivity $\sigma_{dc}$ towards the dispersive region occurs. The permittivity (*ε*) represents the ceramic’s ability to permit the flow of electric field at low frequencies, while the resistivity (*ρ*) indicates the resistance of the ceramic. Observations suggest that as the Cu content increases (resulting in a decrease in resistivity, *ρ*), there is a corresponding shift in the threshold frequency. This shift denotes the point at which the transition from the plateau-type region of $\sigma_{dc}$ towards the dispersive region occurs, and it moves towards higher frequency values.

**Figure 9:**
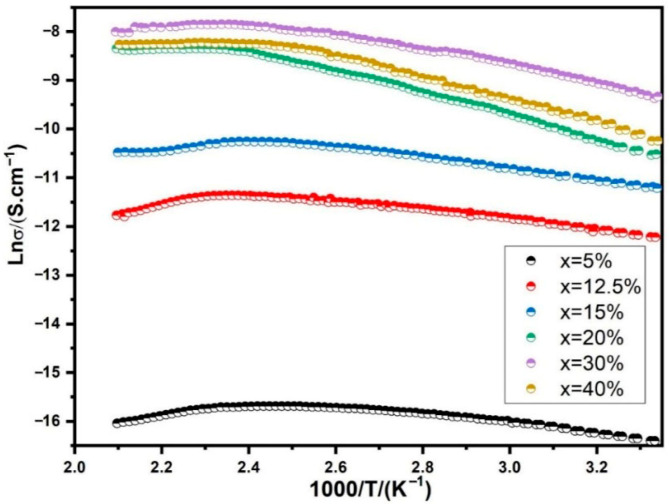
The temperature dependence of the conductivity for Cu-added BST composites.

The temperature dependence of conductivity in BST-Cu is illustrated in Figure 9 for different Cu additions. The graph further presents Arrhenius plots depicting the temperature dependency of conductivity specifically for BST-Cu composites with x = 30 wt%. Below the critical temperature (T_c_) at 130 °C, the conductivity increases with rising measuring temperature, which is characteristic of semiconducting materials. Conversely, the conductivity decreases above Tc, aligning with the behavior typical of conducting materials. This shift in conductivity is attributed to the distinct electrical conduction mechanisms inherent in semiconductors and conductors. As is widely known, semiconductors facilitate electrical conduction by allowing electrons to migrate from the valence band across the forbidden energy gap to the conduction band. Increasing temperature supplies the necessary energy for electrons to traverse the band, enhancing conductivity. In metals, electrons exhibit directional movement, forming electricity. However, at higher temperatures, the electrons experience more intense scattering by metal atoms or ions, hindering their movement and causing a decrease in metal conductivity.

The transformation temperature, approximately Tc, indicates a notable change in the composite’s conductivity-temperature relationship, possibly linked to the ferroelectric-paraelectric phase transition. During this transition, a shift in carrier mobility occurs, resulting in a transformation in conductivity near Tc. The significance of phase change, rather than adhering to a semiconductor-metal model, is emphasized in the BST-Cu composite. This perspective is supported by the well-known observation of conductivity abnormalities around Tc, which is evident in pure BST. Consequently, the phase change holds greater importance than the semiconductor-metal model in understanding the behavior of the BST-Cu composite.

The relationship between conductivity and temperature below 130 °C adheres to an Arrhenius-type correlation, as depicted in Figure 9:

(2) $$\sigma =\sigma_{0}exp(-\frac{E_{a}}{K_{B.T}})$$

Here, *E_a_* represents the activation energy of conduction, *K_B_* is Boltzmann’s constant, *T* is the absolute temperature, and $\sigma_{0}$ is the pre-exponential factor. Consequently, the activation energies of 0.18 eV (below 83 °C) and 0.13 eV (83–130 °C) are significantly lower than the intrinsic bandgap of BST (~3.1 eV), indicating that charge transport is dominated by extrinsic mechanisms such as oxygen-vacancy–assisted hopping and small-polaron conduction between Ti^4+^/Ti^3+^ sites. Because Cu remains as a separate metallic phase and is not incorporated into the BST lattice, Cu-related electronic states cannot account for the activation energy. Instead, the SPS process—conducted under reduced oxygen partial pressure—promotes the formation of oxygen vacancies, which are well-known to create shallow donor states 0.2–0.3 eV below the conduction band, consistent with our measured activation energies [36]. The BST-Cu composites in our study were typically sintered using spark plasma sintering. As a result, the sintering process under low oxygen partial pressure likely generated a certain amount of oxygen vacancies.

**Figure 10:**
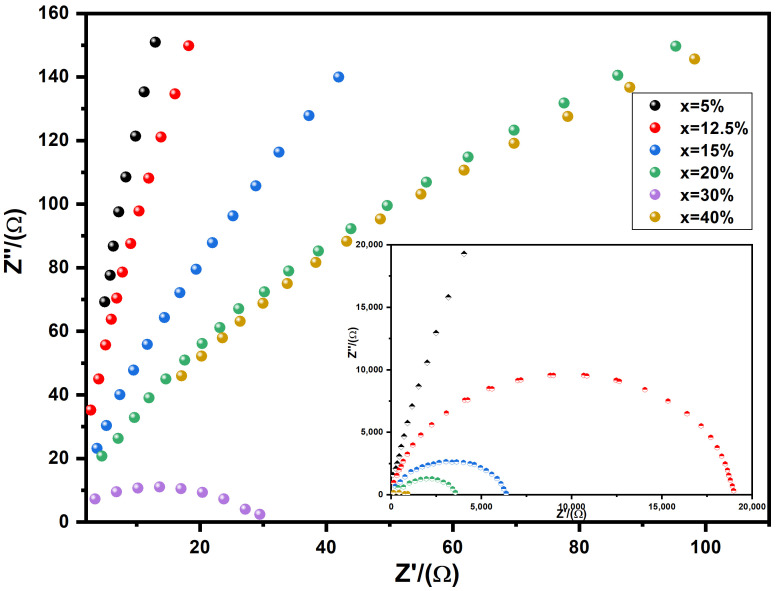
The expanded view of the low frequency and the equivalent circuit of the RC element. The inset represents the impedance complex plane plots for BST-Cux composites of x = 5, 12.5, 15, 20, 25, 30 wt%. The same color samples refer to the same sample names in both the main and inset figures.

Figure 10 depicts complex plane plots for impedance, denoted as Z*, in the BST-Cux composites. These plots illustrate the relationship between the imaginary part (*Z*″) and the real part (*Z*′). Typically, in the case of a flawless crystal, the resistance (*R*) and capacitance (*C*) values are examined using an equivalent circuit consisting of a single parallel RC element. This RC element manifests as a semicircular arc on the complex plane with intercepts at zero and *R* on the *Z*′ axis. Consequently, *C* can be determined using the formula $\omega_{max}$RC = 1, where $\omega_{max}$ = 2πf_max_, and f_max_ represents the frequency at the maximum point of the arc. For BST-Cu composites, the equivalent circuit is conceptualized as two parallel RC elements connected in series, resulting in the appearance of two arcs on the complex plane. One arc corresponds to the grain, and the other corresponds to the grain boundary of the BST-Cu response. The impedance can be calculated using the following approach:

(3) $$Z^{*}=\frac{1}{R_{g}^{-1}+i\omega C_{g}}+\frac{1}{R_{gb}^{-1}+i\omega C_{gb}}=Z^{\prime }-iZ^{\prime \prime }$$

where

(4) $$Z^{\prime }=\frac{R_{g}}{1+{\left(\omega R_{g}C_{g}\right)}^{2}}+\frac{R_{gb}}{1+{\left(\omega R_{gb}C_{gb}\right)}^{2}}$$

And

(5) $$Z^{\prime \prime }=R_{g}\frac{\omega R_{g}C_{g}}{1+{\left(\omega R_{g}C_{g}\right)}^{2}}+R_{gb}\frac{\omega R_{gb}C_{gb}}{1+{\left(\omega R_{gb}C_{gb}\right)}^{2}}$$

**Figure 11:**
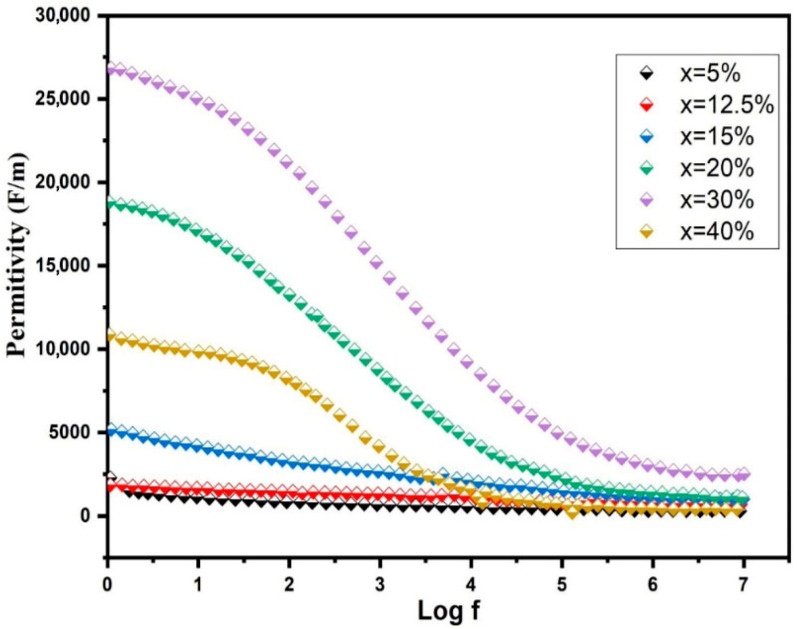
Permittivity of BST-Cu_x_ (x = 5, 12.5, 15, 20, 30, and 40%) composites.

Here, (*R_g_*, *R_gb_*) and (*C_g_*, *C_gb_*) represent the resistance and capacitance values for grains and grain boundaries, respectively. Utilizing Equations (4) and (5), the responses emanating from grains and grain boundaries are positioned at 1/(2π*R_g_C_g_*) and 1/(2π*R_gb_C_gb_*), respectively. The peak value of *Z*″ is directly proportional to the associated resistance. As depicted in Figure 10, it is evident that the *Z*″ peak value attributed to grain boundary responses surpasses that of grain responses (To facilitate visual comparison, different colored circles are employed in Figure 10 to represent the various Cu loadings, illustrating the progressive increase in dielectric permittivity with higher metal content). Consequently, across all examined samples, *R_gb_* is significantly greater than *R_g_*, and the *R_gb_* diminishes with escalating Cu content, aligning with the observations in Figure 8 and Figure 11. These outcomes are elucidated through a two-layer model, postulating the existence of conducting grains isolated by poorly conducting grain boundaries.

Figure 11 presents the frequency-dependent permittivity of BST-Cux composites at room temperature. For x = 5, 12.5, and 15 wt%, a subtle frequency dependency in permittivity was noted within the 100 Hz to 1 MHz range, aligning with the observations in pure BST [37]. As the Cu content increased, reaching x = 30 wt%, the maximum permittivity reached approximately 1.2 × 10^5^ at 1 kHz. However, a noticeable decline in permittivity at low frequencies (around 10^4^ Hz) emerged when x > 30 wt%, indicating a decrease in permittivity at the frequency where tan δ exhibited a relaxation peak. This low-frequency behavior implies the potential influence of conduction mechanisms in the dielectric response of BST-Cu composites. Consequently, the results should be interpreted within the framework of the Maxwell–Wagner capacitor model. In this model, different phases are assumed to possess distinct conductivity, and the overall polarization effects are predominantly influenced by charge accumulation at noncontinuous interfaces within the dielectric. The sensitivity of the Maxwell–Wagner polarization to microstructural heterogeneity is quantified by the broadening of the dielectric loss peaks. As Cu content increases, the distribution of relaxation times (*τ*) widens, reflecting the varied sizes of Cu agglomerates and the presence of BST/Cu/pore triple-junctions. This heterogeneity acts as a site for charge accumulation, where the conductivity mismatch between the metallic Cu (10^7^ S/m) and the BST matrix (10^−8^ S/m) triggers a massive interfacial polarization. However, beyond 30 wt% Cu, the increased porosity and disrupted connectivity of the Cu phase increase the dielectric loss (*tan δ*), as the leakage current paths become dominated by these microstructural defects rather than controlled interfacial storage. The real and imaginary components of relative permittivity and tan δ were computed using the following formula [38],

(6) $$\varepsilon^{\prime }=\varepsilon_{\propto }+\frac{\varepsilon_{0}-\varepsilon_{\propto }}{1+\omega^{2}\tau^{2}}$$

(7) $$\varepsilon^{\prime \prime }=\frac{(\varepsilon_{s}-\varepsilon_{\propto })\omega \tau }{1+\omega^{2}\tau^{2}}+\frac{1}{\omega C_{0}(R_{1}+R_{2})}$$

(8) $$tan\delta =\frac{(\varepsilon_{s}-\varepsilon_{\propto })\omega \tau }{\varepsilon_{s}+\varepsilon_{\propto }\omega^{2}\tau^{2}}+\frac{\sigma }{\omega \varepsilon_{0}}(\frac{1}{\varepsilon_{0}+\frac{\varepsilon_{s}-\varepsilon_{\propto }}{1+\omega^{2}\tau^{2}}})$$

In the given equation, $\varepsilon_{s}$ and $\varepsilon_{\propto }$ represent the static and high-frequency permittivity, respectively. *τ* denotes the relaxation time of the composites, *ω* is the angular frequency, *C*_0_ signifies the capacitance of the empty cell, *R*_1_ and *R*_2_ stand for the resistances of the two dielectric components, *r* represents the conductivity of the composites, and $\varepsilon_{0}$ corresponds to the vacuum permittivity. Equation (6) mirrors the structure of the Debye relaxation equation, stated as follows [39],

(9) $$\varepsilon^{\prime }=\varepsilon_{\propto }+\frac{\varepsilon_{0}-\varepsilon_{\propto }}{1+\omega^{2}\tau^{2}}$$

(10) $$\varepsilon^{\prime \prime }=\frac{(\varepsilon_{s}-\varepsilon_{\propto })\omega \tau }{1+\omega^{2}\tau^{2}}$$

(11) $$tan\delta =\frac{(\varepsilon_{s}-\varepsilon_{\propto })\omega \tau }{\varepsilon_{s}+\varepsilon_{\propto }\omega^{2}\tau^{2}}$$

Evidently, $\varepsilon^{\prime \prime }$ and tanδ in the Maxwell–Wagner model, the deviation from Debye behavior. Examining imaginary permittivity and *tan δ* in the composites reveals distinctions between Debye and Maxwell–Wagner behaviors. Debye relaxation solely accounts for the impact of relaxation polarization on dielectric loss, as evident in the inset illustration of Figure 11, where permittivity sharply diminishes with increasing frequency in the low-frequency range. Simultaneously, a peak in dielectric loss occurs at this frequency, indicating the dispersion frequency. The onset of Maxwell–Wagner behavior in BST-Cu composites suggests that the observed increase in measured permittivity is due to enhanced interfacial polarization rather than relaxation polarization. However, in the Maxwell–Wagner mechanism, the effect of conductance loss (the $\varepsilon^{\prime \prime }$ in Equation (7), which comprises both relaxation polarization and conductance loss), the dielectric loss of composites has been taken into consideration.

Furthermore, the conductivity of BST–Cu composites increases with a rise in Cu content. If Cu were to act as a dopant within the BST lattice, one would expect a reduction in the concentration of conduction electrons due to trapping; however, our observations of increased conductivity confirm that this is a BST/Cu composite system where behavior is governed by interfacial effects. However, our observations indicate the opposite. Instead of being trapped solely in singly ionized oxygen vacancies, electrons may also undergo capture by T^i4+^ in BST, leading to the formation of Ti^3+^. A substantial presence of Ti^3+^ ions on Ti^4+^ sites could result in the hopping of small polarons. Furthermore, SEM observations reveal that at Cu contents ≥40 wt%, Cu particles increasingly agglomerate into isolated clusters rather than forming a continuous conductive network. This microstructural evolution disrupts effective percolation pathways and increases porosity, explaining the observed reduction in conductivity beyond 30 wt% Cu. Thus, the electrical behavior is governed by a balance between Cu connectivity and interfacial continuity.

Nevertheless, the reported activation energy for the hopping of small polarons between Ti^4+^ and Ti^3+^ ions in reduced BST falls within the range of 0.068–0.074 eV [40,41], values significantly lower than those observed in BST–Cu composites. Slight polaron hopping could be a conduction mechanism for analogous BaTiO_3_–Ni composites [42]. The apparent contrast in electrical conduction between BST–Cu and –Ni composites might stem from differences in the concentration of oxygen vacancies and electrons formed through distinct sintering conditions. High permittivity at x = 30 wt% is accompanied by increased losses, indicating an interface-controlled dielectric response. While the BST-Cu30% composite exhibits a giant permittivity of ~10^5^ at 1 kHz, it is accompanied by a significant dielectric loss (tan δ~0.8 at 1 kHz). This behavior is characteristic of Maxwell–Wagner polarization in ceramic-metal systems, where the massive accumulation of charge at the BST/Cu interfaces is inherently linked to leakage currents across the semi-conductive matrix. Frequency-dependent analysis shows that tan δ increases at lower frequencies, confirming that DC conduction and space-charge migration are the primary loss mechanisms. From the perspective of Multi-Layer Ceramic Capacitor (MLCC) relevance, these high losses present a challenge for standard energy storage applications. However, the observed ‘giant’ response demonstrates the effectiveness of Cu-mediated interfacial engineering. For practical MLCC integration, future optimization—such as the implementation of insulating oxide shells around the Cu particles—would be necessary to suppress leakage while maintaining the high interfacial capacitance demonstrated in this study.

To contextualize the technological relevance of the current BST-Cu composites, Table 5 compares the dielectric properties of our optimized BST-Cu30% sample with state-of-the-art BST ceramics and similar metal-reinforced composites. While the dielectric loss (tan δ) in our system is higher than that of commercial BaTiO_3_-based multilayer capacitors, the observed permittivity (~1.2 × 10^5^) is two orders of magnitude higher than pure BST bulk ceramics sintered by conventional methods. This giant dielectric response, driven by Maxwell–Wagner polarization at the BST/Cu interfaces, suggests that these composites are specifically suited for applications requiring high charge storage density in low-frequency decoupling capacitors or pulse-power systems, rather than high-frequency microwave applications where low loss is paramount.

## 4. Conclusions

Ba_0.85_Sr_0.15_TiO_3_–Cu ceramic–metal composites were successfully fabricated via sol–gel synthesis followed by spark plasma sintering, enabling controlled microstructure and multifunctional property tuning. Structural analyses confirmed that Cu remains as a secondary metallic phase without incorporation into the BST lattice or formation of reaction products. The introduction of Cu promotes grain coarsening through localized liquid-phase-assisted sintering while progressively modifying the interfacial microstructure. Optical characterization revealed a pronounced reduction in the effective optical absorption edge (from 3.10 eV to 2.01 eV) with increasing Cu content, arising from interfacial electronic states, defect-related absorption, and enhanced scattering rather than intrinsic band-structure modification. Electrical measurements revealed a percolation threshold at approximately 30 wt% Cu, where both conductivity and dielectric permittivity are maximized. Impedance spectroscopy revealed that the enhanced dielectric response is primarily driven by Maxwell–Wagner interfacial polarization, as confirmed by quantitative equivalent-circuit analysis. Beyond the percolation threshold, Cu agglomeration and porosity disrupt conductive pathways, leading to reduced electrical performance. Overall, this study highlights the critical role of interfacial engineering and percolation control in tailoring the optical and dielectric properties of BST–Cu composites. The optimized compositions offer a viable route for interface-driven high-capacitance devices, provided that future refinements address the high dielectric loss inherent in these percolation-proximate systems. Future work will focus on extending frequency-dependent dielectric analysis and optimizing processing windows to further improve performance stability.

## Figures and Tables

**Table 1:** Specification of chemicals used in sol–gel experiments.

Chemical	Chemical Formula	Company	Purity (%)
Barium acetate	(CH_3_COO)_2_Ba	Sigma–Aldrich	≥99.00
Strontium acetate	(CH3COO)_2_Sr	Sigma–Aldrich	≥99.00
Titanium (IV) isopropoxide	Ti [OCH(CH_3_)_2_]_4_	Sigma–Aldrich	≥97.00
Acetic acid	CH3COOH	Sigma–Aldrich	≥99.50
Acetyl–acetone	CH_3_COCH_2_COCH_3_	Sigma–Aldrich	≥99.30
Distilled water	H_2_O		

**Table 2:** The average Grain size of the as-prepared composites.

Samples	Grain Size (µm)
BST	3.1
BST-Cu5%	3.4
BST-Cu12.5%	3.9
BST-Cu15%	4.1
BST-Cu20%	4.5
BST-Cu30%	4.7
BST-Cu40%	5.2

**Table 3:** Quantitative elemental composition and stoichiometric ratios of Ba_0.85_Sr_0.15_TiO_3_/Cu composites determined by EDS point analysis.

Specimen	Ba (at.%)	Sr (at.%)	Ti (at.%)	A-Site Sum (Ba + Sr)	O (at.%)	Cu (at.%)	O/Ti Ratio
Pure BST	16.98 ± 0.1	3.02 ± 0.1	20.02 ± 0.2	20.00	59.98 ± 0.5	—	2.99
BST–Cu15%	14.16 ± 0.2	2.51 ± 0.1	16.68 ± 0.3	16.67	50.01 ± 0.6	16.64 ± 0.4	3.00
BST–Cu40%	10.11 ± 0.2	1.80 ± 0.1	11.92 ± 0.3	11.91	35.79 ± 0.7	40.38 ± 0.5	3.01

**Table 4:** Band gap value of BST-Cu_x_ (x = 0–40%).

Sample (% wt.)	Band Gap Value (eV)
x = 0.00	3.10
x = 5	2.93
x = 12.5	2.85
x = 15	2.72
x = 20	2.31
x = 30	2.21
x = 40	2.01

**Table 5:** Comparison of dielectric permittivity and loss tangent of the optimized BST–Cu30% composite with various state-of-the-art BST-based ceramics and metal–ceramic composites.

Material System	Permittivity (ϵr)	Loss (tan δ)	Polarization Mechanism	Primary Application
Pure BST (This study)	~1200	0.02	Dipolar	Standard MLCCs
BST–Cu30% (This study)	~120,000	0.80	Maxwell–Wagner	High-C Decoupling
BaTiO_3_–Ni [43]	~10,000–50,000	0.5–1.0	Percolation/Interfacial	Energy Storage
BST Bulk [44]	~1500–3000	0.01–0.05	Dipolar	Capacitors/Sensors
BST Thin Films [45]	~500–1000	<0.01	Intrinsic/Domain	Microwave Tunable

## Data Availability

The data supporting this study’s findings are available from the corresponding author upon reasonable request.
